# The Hsp70 Gene Family in *Boleophthalmus pectinirostris*: Genome-Wide Identification and Expression Analysis under High Ammonia Stress

**DOI:** 10.3390/ani9020036

**Published:** 2019-01-26

**Authors:** Zhaochao Deng, Shanxiao Sun, Tianxiang Gao, Zhiqiang Han

**Affiliations:** Fishery College, Zhejiang Ocean University, Zhoushan 316002, Zhejiang, China; 18767788185m@sina.cn (Z.D.); jxmjsx@163.com (S.S.); gaozhang@ouc.edu.cn (T.G.)

**Keywords:** *Boleophthalmus pectinirostris*, heat shock proteins 70, high environmental ammonia, genome-wide identification

## Abstract

**Simple Summary:**

Heat shock proteins 70 is a family of proteins, which were expressed in response to a wide range of biotic and abiotic stressors. The development of genomic resources and transcriptome sequences makes it practical to conduct a systematic analysis of these genes. In this study, exhaustive searches of all genomic resources for *Boleophthalmus pectinirostris* Hsp70 genes were performed and their responses to high environmental ammonia stress were investigated. Besides, selection test was implemented on those duplicated genes, and the phylogenetic tree, gene structure, and motif analysis were also constructed to assign names of them. The result showed that there were 20 Hsp70 genes within the genome of *Boleophthalmus pectinirostris*, and some sites in the duplicated genes may experience positive selection, and most of Hsp70 genes were downregulated after exposure to high concentration ammonia. The present results of this study can be used as a reference for further biological studies on mudskippers.

**Abstract:**

Heat shock proteins 70 have triggered a remarkable large body of research in various fishes; however, no genome-wide identification and expression analysis has been performed on the Hsp70 gene family of *Boleophthalmus pectinirostris*. In this study, we identified 20 Hsp70 genes within the genome of *B. pectinirostris* and provided insights into their response to high environmental ammonia (HEA) stress. Positive selection on stress response genes and expansion of *hspa1a* and *hspa1a*-like genes might be related to terrestrial adaptations in this species. The expression patterns of the Hsp70 gene family in the gill and liver of *B. pectinirostris* under HEA stress were studied by examining transcriptome data. The results showed that most Hsp70 genes were downregulated after high concentration ammonia exposure. The downregulation may be related to the hypoxic condition of the tissues.

## 1. Introduction

Heat shock proteins (Hsps) are a large group of molecular chaperones. The expressions of this gene family are induced by a wide range of biotic and abiotic stressors, some members are constitutively expressed in non-stressed cells served as housekeeping proteins [1]. The high degree of identity on the amino acid sequence and their essential physiological roles in nearly all organisms, make this group of proteins unique [2]. Based on their molecular weight, Hsps are classified into Hsp110, Hsp90, Hsp70, Hsp60, Hsp40, Hsp10, and small Hsps [3]. Among them, the Hsp70 gene family is the most extensively studied group of HSPs. 

Plenty of evidence has reported that the expression patterns of Hsp70 genes can be induced by various environmental stress factors [4,5,6] and they play essential roles, not only in protein folding, membrane translocation, degradation of misfolded proteins and other processes for the protection of cells, but also in responses to stress, bacterial infections, parasitism and inflammation [7,8]. A potent buffering system were supplied by Hsp70 gene family for protection of cellular stress, either from extrinsic (physiological, pathogenic, or environmental) or intrinsic (replicative or oncogenic) stimuli [3].

The members of the Hsp70 gene family have been systematically characterized in some species of fungus [9,10], plants [11,12,13], and mammals [14,15]. Although they have also triggered a remarkably large body of research in various fishes, only a few studies have focused on expression profiles and genome-wide identification of this gene family, such as studies investigating *Fugu rubripes* [16] and channel catfish [1]. The current lack of genomic resources and transcriptome sequences in fishes is likely responsible for this research gap. In addition, the use of different names for the same Hsp70 gene or protein and the use of the same name for various Hsp70 genes or proteins have generated confusion in literatures. It is hard to understand which gene or protein in the family is referred to, when the term Hsp70 is cited without further description. 

Ammonia is a major environmental pollutant [17]. It can come from industrial wastes, household waste, agricultural run-off, and decomposition of natural biological waste [18]. Meanwhile, it is the main nitrogenous excretory product of bony fish (teleosts), which accumulates easily in aquaria and aquaculture systems [8,19]. High environmental ammonia (HEA) causes oxidative stress in aquatic animals through increasing the concentration of reactive oxygen species (ROS) [20,21], resulting in the loss of cellular membrane integrity, extensive damage of DNA and cellular apoptosis [22]. Although the Hsp70s play important roles in repairing and clearance of damaged proteins under various stress including the oxidative stress [23,24], few studies have analyzed the Hsp70 gene expression of fish species in response to HEA. 

*Boleophthalmus pectinirostris*, an amphibious Gobiidae fish, mainly inhabits in the mud flat around river mouths along the coast of eastern Asia [25]. It occasionally faces the problem of HEA when it is trapped in puddles of water. As a result, *B. pectinirostris* is an ideal species for expression analysis of the Hsp70 gene family in response to HEA. Previous studies have shown one member of the Hsp70 gene family of *B. pectinirostris* playing an important role in protection against heat stress [26], and transcriptomic evidence of adaptive tolerance to HEA in *B. pectinirostris* [27]. However, there is still no genome-wide identification of this gene family in this species. The genomic resources and transcriptome sequences of *B. pectinirostris* have been provided in recent years [28], which makes it feasible to conduct a systematic analysis of these genes in the *B. pectinirostris* genome.

In the present study, a genome-wide identification of a full set of Hsp70 genes in *B. pectinirostris* was conducted, and their gene expressions under HEA stress were investigated. Twenty Hsp70 genes were reported in the genome of *B. pectinirostris*. Their phylogenetic relationship, gene structure, conserved domain, and expression profiles in response to HEA were analyzed. The findings of this study may help illuminate the regulatory mechanism of the Hsp70 gene family in response to environmental stress and provide useful resources for future studies of Hsp70 genes; it will also facilitate the study of Hsp70 genes in different fish species.

## 2. Materials and Methods 

The genome sequence, transcriptome sequences, and protein sequences of *B. pectinirostris* were downloaded from NCBI databases (JACK00000000.1) [28]. Two strategies for identifying the full set of Hsp70 genes in the *B. pectinirostris* genome were used. First, Blastp (standard protein BLAST) searches were performed against amino acid sequences of *B. pectinirostris* using Hsp70s identified from humans and zebrafish as query sequences. Second, a hidden Markov model (HMM) profile of the Hsp70s was employed to query the *B. pectinirostris* dataset using HMMER software [29,30]. The HMM profile was downloaded from the Pfam protein family database (version 32, http://pfam.xfam.org/), whereas the HMM profiles of Hsp12a and Hsp12b (PTHR14187:SF46 and PTHR14187:SF39) were obtained from the Protein Analysis Through Evolutionary Relationships Classification System (PANTHER version 14.0, http://www.pantherdb.org/). The e-value was set at an intermediately stringent level of e^−10^ to collect candidate Hsp70s-related sequences for further analysis. The online program Pfam (version 32, http://pfam.xfam.org/search) and the Conserved Domain Database from NCBI (CDD) (version 3.16, http://www.ncbi.nlm.nih.gov/structure/cdd) were used to survey the conserved domains of the candidate proteins. Furthermore, the obtained full conserved domain sequence (CDS) of proteins from the *B. pectinirostris* genome were used as queries to search against this species in RNA-Seq datasets. Moreover, to distinguish which of the Hsp70 genes are Hsf-induced (contain a heat shock element) in *B. pectinirostris*, the raw HMM and protein sequences of Hsf were downloaded to find their binding sites and were compared to the locus of Hsp70 genes in the genome.

The protein sequences of Hsp70 genes identified from *B. pectinirostris*, humans (*Homo sapiens*), Nile tilapia (*Oreochromis niloticus*), zebrafish (*Danio rerio*), medaka (*Oryzias latipes*), and Stickleback (*Gasterosteus aculeatus*) were used to construct a phylogenetic tree. The protein sequences of these species were retrieved from the NCBI (http://www.ncbi.nlm.nih.gov), UniProt (version 2018_01, http://www.uniprot.org), and Ensembl (http://asia.ensembl.org/index.html) databases [1] (Table S1). We used the MUSCLE method in the program MEGA 6.0 to conduct the multiple protein sequence alignments [31]. We constructed the neighbor joining (NJ) tree in MEGA 6.0 software with the complete deletion option. Bootstrap analyses were used with 1000 replicates to assess the support for phylogenetic relationships. 

The gene structure of *B. pectinirostris* Hsp70 genes was analyzed using TBtools software version 0.66 [32] based on the genome annotation file. The conserved DNA sequence motifs in the Hsp70s were determined by Multiple Expectation Maximization for Motif Elicitation (MEME) software (version 5.0.2) [33] according to the following parameters: site distribution was set at 0 or 1 occurrence per sequence, the number of motifs found to be more suitable was 15, and the motif width was set between 18 and 150. The outputs generated by MEME was used to GOMo scans (Gene Ontology for Motifs) that can suggest the biological roles of the motifs [34].

The RNA-Seq data were retrieved from HEA challenge experiments of *B. pectinirostris* (SRR5012115-SRR5012118 in the NCBI database) to study the expression profiles of Hsp70 genes. Six individuals of *B. pectinirostris* exposed to artificial seawater containing 8mM NH4Cl at 27 °C for 72 h were served as the test group, and six individuals of *B. pectinirostris* immersed in artificial seawater at 27 °C were served as the control group. The gills and livers of each fish in each group were collected. Six genes including *rhcg1*, *ca15*, *nhe3*, *alt*, *ass*, and *me* were used for the validation of transcriptome data by quantitative real time PCR (qRT-PCR) [28].

The differential gene expressions were analyzed using RSEM (expectation maximization). The expression estimations of Hsp70 genes were represented and normalized in the form of fragments per kilobase of exon per million fragments mapped (FPKM), and log2 based fold change (log_2_FC) values were calculated. Genes with a log_2_FC > 1.5 and *t*-test values (*p* < 0.05) were defined as differentially expressed genes. A heat map was also generated using MeV 4.90 software (http://www.tm4.org) with the log_2_FC values.

Based on the phylogenetic relationships and expression profiles of the Hsp70 genes, the CodeML algorithm [35] was implemented to the *hspa1a* and *hspa1a-like* genes in *B. pectinirostris* to examine the specific gene expansion of this species. The non-synonymous substitutions (*dN*)/synonymous substitution (*dS*) value could be used to distinguish among neutral evolution (*dN/dS* = 1), purifying selection (*dN/dS* < 1) and positive selection (*dN/dS* > 1). Two comparisons (M1a versus M2a and M7 versus M8) of site models that allowed the ω ratio to vary among sites were used to detect positive selected sites. The likelihood ratio tests (LRT) with a chi-square distribution were used for comparison of model pairs.

## 3. Results

### 3.1. Genome-Wide Identification of Hsp70 Genes in B. pectinirostris

A total of 23 putative Hsp70 genes were initially obtained from BLAST and HMM searches. Based on the confirmation of Pfam and NCBI CDD scans, three candidate genes (without the Hsp70 domain) were discarded. Twenty residual members symbolizing the unique Hsp70 gene family in *B. pectinirostris* were used to create robust nomenclature following guidelines for the nomenclature of the human heat shock proteins [36]. Detailed information about members of Hsp70 gene family is shown in Table 1. To avoid confusion created by the Hsp70 names and to clarify how the names were denoted to *B. pectinirostris* Hsp70 genes, comparison the nomenclature among human, zebrafish, and *B. pectinirostris* was listed in Table 2. 

Among these genes, full lengths of all coding sequences were detected in both transcriptome and genome databases. Three Hsp70 genes (XM_020937593.1; XM_020934606.1; XM_020934610.1) were identified as ‘heat shock cognate 71 protein’ before, and were renamed as *hspa8*, *hspa8a.1*, and *hspa8a.2* after the phylogenetic tree, respectively. The *hspa14* had the shortest conserved domain with 375 amino acids, whereas the longest domain was found in the *hspa1a* and *hspa1a-like* genes.

Many Hsp genes are bound by *hsf* or *hsf-like* genes and could be induced in an Hsf-dependent manner upon heat shock response. However, only hspa9 gene was found to be bound by *hsf-like* gene in the Hsp70 gene family of *B. pectinirostris*. In other words, the *hspa9* gene was predicted to be Hsf-induced in *B. pectinirostris*.

### 3.2. Phylogenetic Relationships of the Hsp70 Genes among Species

**Figure 1:**
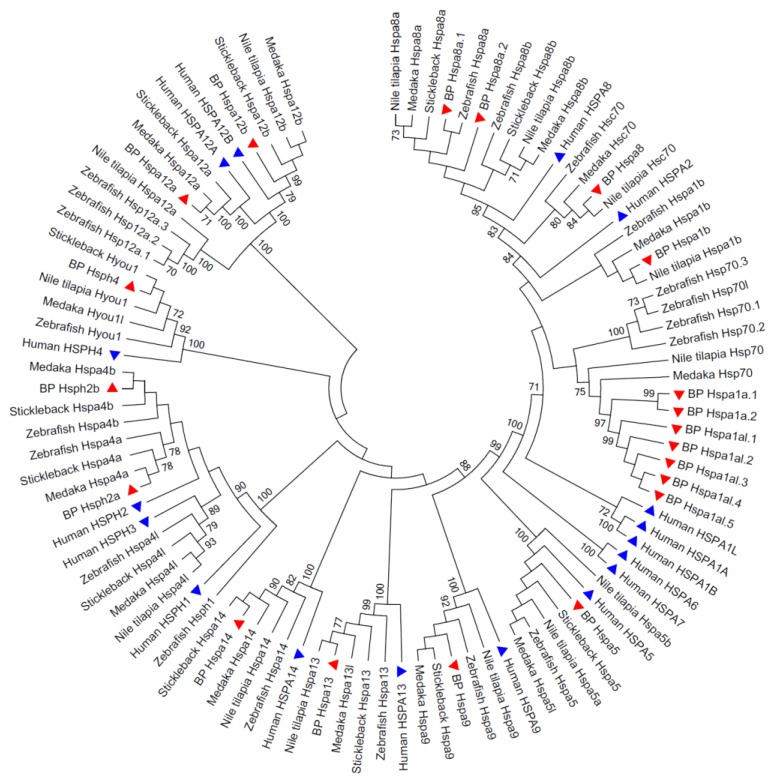
Phylogenetic tree of Hsp70 families from selected organisms. Hsp70s from *B. pectinirostris* and zebrafish are marked with red and blue triangles, respectively. Bootstrap supports of >70% in 1000 replicates are shown.

An unrooted phylogenetic NJ tree was generated using the amino acid sequences of Hsp70s (Figure 1). Names were assigned to each of them based on the clade of the NJ tree. Seven copies of *B. pectinirostris hspa1a* genes (*hspa1a*, *hspa1a-like*) were found to be highly orthologous to the medaka *hsp70* gene, the Nile tilapia *hsp70* gene and the zebrafish *hsp70* and *hsp70-like* genes. Based on previous studies and the phylogenetic tree, the *hspa1* gene in the ancestor of teleosts might be divided into two clades, and the *hsp70* genes appeared earlier than *hspa1b*. As a result, the *hsp70* genes were named *hspa1a* and *hspa1a-like* in *B. pectinirostris* following the guidelines for the nomenclature of the human heat shock proteins. *B. pectinirostris* apparently had more duplicated *hspa1a-like* genes than other teleosts as shown in Figure 1. The *hspa8b* gene was not found in this species, whereas two copies of *hspa8a* were present in the phylogenetic tree. All members of the *B. pectinirostris* Hsp70 gene family were well distributed into distinct groups, first clustered with corresponding genes of other fish species and supported by high bootstrap values (Figure 1). 

Most pairs of orthologs from *B. pectinirostris* and other fish species were presented, suggesting that the common ancestral genes of this gene family might have existed before the speciation of fish species. Several pairs of duplicated Hsp70 genes were found in *B. pectinirostris*, indicating that Hsp70 genes might undergo some duplication events after speciation.

### 3.3. Gene Structure and Motif Analysis of Hsp70s

**Figure 2:**
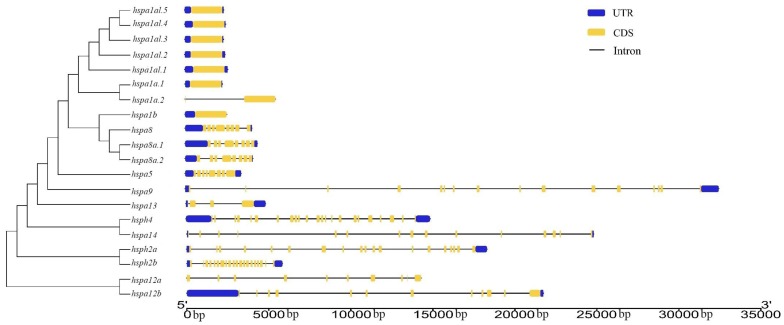
Gene structure analyses of *B. pectinirostris* Hsp70 genes according to phylogenetic relationship. The blue boxes, yellow boxes, and the black lines indicate UTR (untranslated region), exons, and introns, respectively.

Figure 2 provided the organization of the introns and the corresponding exons within each Hsp70 gene in *B. pectinirostris*. Eight Hsp70 gene members had only one exon, whereas other genes had 3–23 exons. The exon numbers frequently varied with phylogenetic relationships among the Hsp70 genes in *B. pectinirostris*, except the *hspa13* gene. The similar structures of the *hspa8a.1* and *hspa8a.2* genes and the structures between *hspa1a* and *hspa1a-like* genes offer a rationale for their standardized names.

**Figure 3:**
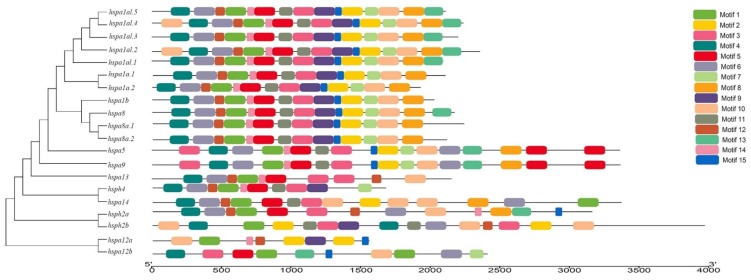
The conserved motifs of *B. pectinirostris* Hsp70s according to phylogenetic relationship. All motifs are identified by MEME database with the complete nucleotide sequences of *B. pectinirostris* Hsp70s. Each colored box represents a motif detected in the corresponding sequence. MEME: Multiple Expectation Maximization for Motif Elicitation.

Conserved motif analysis was performed based on the evolutionary relationships among the complete nucleotide sequences of the *B. pectinirostris* Hsp70s (Figure 3). Fifteen putative motifs were searched for in each Hsp70s as shown in Figure 3. After GOMo search, motifs 1, 2, 3, 9, 10, and 11 were annotated as sequence-specific DNA binding and transcription factor activity motif, and motifs 14 and 15 were annotated as K^+^ and Mg^2+^ potassium transport, respectively. As shown in Figure 3, all the identified Hsp70 genes contained motif 1, and most of them had motifs 3, 10, and 14, which might contribute to the identification of this gene family and understanding of their potential functions. Meanwhile, the Hsp70s from close evolutionary clusters shared similar motifs. The results of the motif analysis provided further support to the phylogenetic classification of Hsp70 gene family.

### 3.4. Expression Profiles of Hsp70 Genes in Different Tissues under HEA Stress

**Figure 4:**
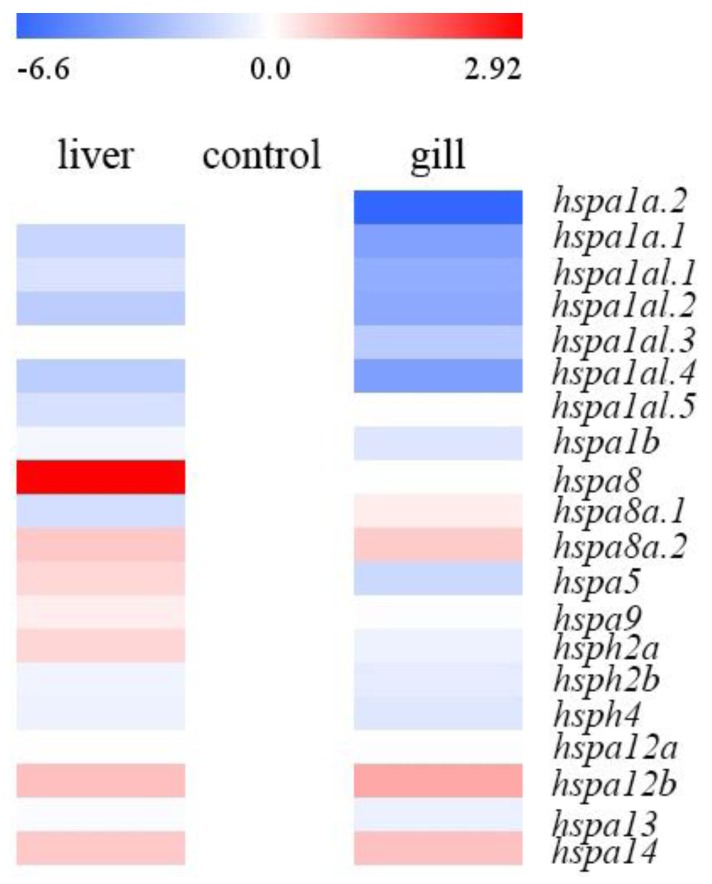
Expression profiles of *B. pectinirostris* Hsp70 genes in different tissues. The expression level of each gene was compared against the data of control (0 h). Log2 based fold change in FPKM values was used to create the heat map. FPKM: fragments per kilobase of exon per million fragments mapped.

To study the expression regulation of Hsp70 genes in different tissues, transcriptome of gills and livers were analyzed (Figure 4; Table 3). As shown in Table 3, 8 out of 20 Hsp70 genes were significantly involved in HEA stress responses (log_2_FC > 1.5 or < −1.5). Among these, three genes were downregulated and only one gene (*hspa8*) was upregulated in the liver, whereas seven genes were strongly downregulated in the gill. In addition, *hspa1a.1*, *hspa1a.2*, and *hspa1al.4* genes were strongly downregulated in both tissues (log_2_FC: −4.14 to −1.75). The *hspa1a.3* and *hspa5* genes were downregulated only in the gill (log_2_FC: −2.21 and −1.61, respectively) after HEA treatment, whereas the *hspa8* gene was upregulated only in the liver (log_2_FC: 2.92). Apparently, the duplicated *hspa1a* genes were more inducible than other genes (Table 3) after HEA stress. As a whole, the genes from the gill were more reactive than those from the liver (Table 3; Figure 4).

### 3.5. Selection on Duplicated Hsp70 Genes

As mentioned above, *B. pectinirostris* had more duplicated *hspa1a* genes and most of them were significantly involved in HEA stress responses in both the gill and the liver. To better understand the species specific gene expansion, codon-based site models of evolution implemented in PAML Version 4.9 (Phylogenetic Analysis by Maximum Likelihood) were used. The results of comparisons of M2a vs. M1a and M8 vs. M7 showed that significance of positive selection existed among these genes (Table 4). The Bayes empirical Bayes (BEB) can be used to identify sites under positive selection if the likelihood ratio test is significant [34]. The BEB showed that several sites were under positive selection in M2a and M8 (Table 4).

## 4. Discussion

In this study, we performed an overall analysis of the Hsp70 gene family in *B. pectinirostris*, including an analysis of phylogeny, gene structure, conserved motifs, expression patterns under HEA stress and selection tests. These information may be useful for genome analysis and annotation as well as for evolutionary studies in fish species.

A total of 20 Hsp70 genes were identified and annotated in this species. Compared with humans and other fish species, most of the Hsp70 gene members were found in *B. pectinirostris* except *hsph1* and *hspa8b*. The absence of the *hsph1* gene in *B. pectinirostris* was consistent with results obtained for catfish [1] and other fish species (not including zebrafish). The *hspa8b* gene was seemingly lost in this species, whereas two repeats of its paralogs *hspa8a* were found. However, it is uncertain whether the *hsph1* and *hspa8b* genes are truly missing from the *B. pectinirostris* genome, although exhaustive searches of all genomic resources for this species were performed. 

It was difficult to assign names to some Hsp70 genes solely by the clade of the phylogenetic tree. Nevertheless, combining the NJ tree with motif analysis, one can easily and accurately distinguish among these genes. This method has also been applied to plants [37,38] and humans [14]. Moreover, the gene structure and the type, order and number of the motif changes may reflect their specific functions that are not shared with other genes. Due to the requirements of specific capabilities, animals under certain environments might duplicate relative genes throughout their population history. 

Gene duplication has been thought to play an important role in species adaptation and could provide raw genetic material for genes with new functions [3,39]. The present study suggests that local gene tandem duplication may be an important mechanism of Hsp70 gene expansion in *B. pectinirostris* since all of the expanded *hspa1a* genes exist as tandem gene clusters (Table 1). Tandem duplications of *hspa1a* (named *hsp70* before) paralogs have previously been described in zebrafish [40], stickleback, and *Tetraodon nigroviridis* [41]. Nevertheless, the number of the *hspa1a* tandemly duplicated genes differs among species, indicating that these tandem duplications may have independent origins. Furthermore, the results of selection tests on these *hspa1a* and *hspa1a-like* genes have shown that some sites may experience positive selection, although this is rare (with a proportion of about 0.73%) (Table 4), supporting that these genes undergo sub-functionalization or acquire new functions in the *B. pectinirostris*. As is known, *B. pectinirostris* is a typically amphibious teleost fish, and the water-to-land transition must lead to the emergence of high environmental stress. Previous studies have shown a significant increase on expression levels of *hspa1a* genes in other species under severe environment, such as medaka under high temperature [4], *Monopterus cuchia* under high ammonia stress [24], and *Umbra limi* under exposure to the air [42]. It is reasonable to assume that the stress response genes and expansion of *hspa1a* and *hspa1a-like* genes might have evolved terrestrial adaptations in the *B. pectinirostris*, which enabled them to spend a considerable part of their lives on land. Previous studies have shown three different expression patterns under thermal stress in mammalian HSP70 gene family [14,43]: (A) strictly heat-inducible HSP70; (B) cell-cycle-dependent and heat-inducible HSP70; and (C) constitutively expressed and less stress-dependent HSP70 genes. In this study, *hspa1a.2*, *hspa1al.3*, *hspa1al.5*, *hspa1b*, *hspa12a*, and *hspa12b* genes were expressed constitutively at very low levels, and *hspa8a.2*, *hspa9*, *hsph2a*, *hsph2b*, *hsph4*, *hspa13*, and *hspa14* genes—all of which should be put into group C—were expressed constitutively but scarcely induced by HEA stress. Other Hsp70 genes—including *hspa1a.1*, *hspa1al.1*, *hspa1al.2*, *hspa1al.4*, *hspa8*, *hspa8a.1*, and *hspa5*, which were put into group B—were relatively highly expressed or showed significantly different expression levels between test and control. There was no most inducible gene response to HEA stress. Different expression patterns were also found in Hsp70 genes of channel catfish after bacterial infection [1]. Hence, the present study focused on group B genes to study the expression regulation of Hsp70 genes in different tissues of *B. pectinirostris* in response to HEA stress. Six Hsp70 genes of group B were significantly up- or downregulated after HEA stress, indicating their involvement in stress responses. Three genes were downregulated and only 1 was upregulated in the liver, whereas five genes were strongly downregulated and none were significantly upregulated in the gill. The genes from the gill were apparently more reactive than those from the liver. This may be because excretion of ammonia occurs mainly through the gills of fishes [27], and gills are well known as the first guard to react to unfavorable environmental conditions [44]. Moreover, some Hsp70 genes were expressed in only one tissue, showing a tissue-specific pattern. 

Because the concentration of ammonia applied in this experiment only induced physiological stress but did not result in the death of individuals [45], and when we took the function of the Hsp70 genes into consideration, it was unexpected that most of them were downregulated in both tissues in this study. Previous studies have shown that *B. pectinirostris* can decrease the production rate of ammonia from amino acid catabolism in hepatocytes [46] to slow down the build-up of internal ammonia [47] under ammonia exposure. The protein digestion-related genes were downregulated, directly or indirectly, causing the expression of most Hsp70 genes to decline in the liver after HEA stress. 

However, it was difficult to explain the expression pattern in the gills. One plausible explanation for downregulated expression was that a hypoxic condition might be generated via the exposure to elevated NH_3_ levels [48], and the hypoxia of tissues might restrain the expression of Hsp70 genes. To some extent, this speculation was supported by studies examining the toxicology of ammonia nitrogen, which showed that the ammonia toxicity increased as the concentrations of dissolved oxygen (DO) decreased, whereas the resilience of aquatic organisms to environmental ammonia stress can be improved when the concentrations of DO increased [49,50]. To further verify the hypothesis, the transcriptome data of the brain in the large yellow croaker (*Larimichthys crocea*) under hypoxia stress was downloaded from NCBI (accession number: SRX541138, SRX541136, and SRX541132) and was re-analyzed. When the 48-h test group was compared with the 0-h group, almost all of the expression profiles of Hsp70 genes were downregulated in the brain of the large yellow croaker under hypoxia stress [51] (Figure S1), which also supported the hypothesis of the present study. The expression patterns of Hsp70 genes under HEA and hypoxia stresses showed that the functions of this gene family might require sufficient oxygen. The other possibility for the downregulated expression was that Hsp70 genes could not be triggered by high ammonia concentration in this species under the provided experimental condition. As many Hsp70 genes are induced primarily upon acute protein-damaging conditions [52], however, *B. pectinirostris* possesses a greater capacity to detoxify ammonia and high tolerance to environmental ammonia [28], which might make ammonia fail to induce acute protein-damaging stress. 

## 5. Conclusions

In the present study, we identified 20 Hsp70 genes within the genome of *B. pectinirostris* and provided insights into their response to high environmental ammonia (HEA) stress. This study may contribute to illuminate the regulatory mechanism of the Hsp70 gene family in response to environmental stress. Certainly, due to the absence of data from other time periods (such as 12 h or 48 h), the deficiency of the expression profiles of Hsp70 genes under both HEA stress and high concentrations of DO, and a general lack of biological and physiological knowledge, further verifications are needed to predict the exact implications of the changes.

## Figures and Tables

**Table 1:** Summary of 17 Hsp70 genes identified in genome of *B. pectinirostris.*

No.	Gene Name	Gen Accession Number	Protein Accession Number	CDS * Length (bp)	Protein Length (aa)	Hsp70 Domain Location (aa)	Domain Feature
1	*hspa1al.5*	XM_020933256.1	XP_020788915.1	1917	638	2-638	HSPA1-2_6-8-like_NBD
2	*hspa1al.4*	XM_020933257.1	XP_020788916.1	1917	638	2-638	HSPA1-2_6-8-like_NBD
3	*hspa1al.1*	XM_020933255.1	XP_020788914.1	1917	638	2-638	HSPA1-2_6-8-like_NBD
4	*hspa1al.2*	XM_020933253.1	XP_020788912.1	1917	638	2-638	HSPA1-2_6-8-like_NBD
5	*hspa1al.3*	XM_020933254.1	XP_020788913.1	1917	638	2-638	HSPA1-2_6-8-like_NBD
6	*hspa1a.1*	XM_020932913.1	XP_020788572.1	1917	638	2-638	HSPA1-2_6-8-like_NBD
7	*hspa1a.2*	XM_020933010.1	XP_020788669.1	1920	639	4-639	HSPA1-2_6-8-like_NBD
8	*hspa1b*	XM_020929942.1	XP_020785601.1	1917	638	2-638	HSPA1-2_6-8-like_NBD
9	*hspa8*	XM_020937593.1	XP_020793252.1	1968	655	1-612	HSPA1-2_6-8-like_NBD
10	*hspa8a.1*	XM_020934606.1	XP_020790265.1	1953	650	1-614	HSPA1-2_6-8-like_NBD
11	*hspa8a.2*	XM_020934610.1	XP_020790269.1	1953	650	1-613	HSPA1-2_6-8-like_NBD
12	*hspa5*	XM_020925086.1	XP_020780745.1	1956	651	28-633	HSPA5-like_NBD
13	*hspa9*	XM_020929023.1	XP_020784682.1	2043	680	53-658	HSPA9-like_NBD
14	*hspa13*	XM_020926778.1	XP_020782437.1	1314	437	12-429	HSPA13-like_NBD
15	*hspa14*	XM_020940290.1	XP_020795949.1	1524	507	2-376	HSPA14-like_NBD
16	*hsph2a*	XM_020925033.1	XP_020780692.1	2508	835	2-384	HSPA4_NBD
17	*hsph2b*	XM_020927815.1	XP_020783474.1	2487	828	2-384	HSPA4_NBD
18	*hsph4*	XM_020934583.1	XP_020790242.1	2928	975	30-416	HYOU1-like_NBD
19	*hspa12b*	XM_020920454.1	XP_020776113.1	2091	696	68-535	HSPA12B_like_NBD
20	*hspa12a*	XM_020937725.1	XP_020793384.1	1549	515	1-379	HSPA12A_like_NBD

* CDS: Coding sequence.

**Table 2:** HSP70 gene families in human, zebrafish, and *B. pectinirostri*.

No.	*Homo sapiens*	*Danio rerio*	*Boleophthalmus pectinirostris*
1	*HSPA1A*	*hsp70.1; hsp70.2; hsp70.3; hsp70-like*	*hspa1a.1; hspa1a.2; hspa1al.1; hspa1al.2; hspa1al.3; hspa1al.4; hspa1al.5*
2	*HSPA1B*	*hspa1b*	*hspa1b*
3	*HSPA1L*	*-*	*-*
4	*HSPA2*	*-*	*-*
5	*HSPA5*	*hspa5*	*hspa5*
6	*HSPA6*	*-*	*-*
7	*HSPA7*	*-*	*-*
8	*HSPA8*	*hspa8a; hspa8b*; *hsc70*	*hspa8a.1; hspa8a.2*; *hspa8*
9	*HSPA9*	*hspa9*	*hspa9*
10	*HSPA12A*	*hspa12a.1; hspa12a.2; hspa12a.3*	*hspa12a*
11	*HSPA12B*	*-*	*hspa12b*
12	*HSPA13*	*hspa13*	*hspa13*
13	*HSPA14*	*hspa14*	*hspa14*
14	*HSPH1*	*hsph1*	*-*
15	*HSPH2*	*hspa4a; hspa4b*	*hsph2a; hsph2b*
16	*HSPH3*	*hspa4l*	*-*
17	*HSPH4*	*hyou1*	*hsph4*

**Table 3:** Log_2_ based fold change (log_2_FC) and the FPKM of *B. pectinirostris* Hsp70 gene expression in liver and gill at 0 h and after 72 h of HEA stress. The significant genes (*p* value < 0.05, total reads number > 10, log_2_FC > 1.5) are in bold. The FPKM for expression estimations of different tissues are in shading.

Gene\Tissue	Liver			Gill		
	log_2_FC	0 h	72 h	log_2_FC	0 h	72 h
*hspa1a.2*	-	0	0	**−6.6**	1.94	0.02
*hspa1a.1*	**−1.75**	7.68	2.29	**−4.02**	20.63	1.27
*hspa1al.1*	−1.2	49.14	21.39	**−3.54**	37.57	3.23
*hspa1al.2*	**−2.17**	59.1	13.13	**−3.72**	64.8	4.91
*hspa1al.3*	-	0	0.63	**−2.21**	1.62	0.35
*hspa1al.4*	**−2.13**	54.67	12.45	**−4.14**	75.78	4.31
*hspa1al.5*	−1.28	8.48	3.49	-	0	0.67
*hspa1b*	−0.32	1.24	0.99	−1.04	1.46	0.71
*hspa8*	**2.92**	3.06	23.11	0.01	23.32	23.51
*hspa8a.1*	−1.35	3483.05	1368.19	0.22	2486.01	2889.56
*hspa8a.2*	0.64	14.15	22.14	0.61	38.26	58.47
*hspa5*	0.46	227.06	312.16	**−1.61**	363.63	118.77
*hspa9*	0.21	48.79	56.41	−0.03	60.68	59.34
*hsph2a*	0.48	3.53	4.92	−0.52	9.02	6.27
*hsph2b*	−0.45	20.68	15.15	−0.77	64.33	37.78
*hsph4*	−0.54	29.33	20.23	−1	21.98	11.01
*hspa12a*	-	0.05	0.06	-	0.41	0.27
*hspa12b*	0.74	0.37	0.62	0	2.91	2.91
*hspa13*	−0.11	14.39	13.32	−0.58	16.85	11.24
*hspa14*	0.62	15.69	24.16	0.73	29.67	49.24

**Table 4:** Parameters, positively selected sites, and hypothesis test under site model.

Model	−InL *^a^*	2ΔInL	df	Parameters	Positively Selected Sites
M1a	3122.75	-	-	p0 = 0.9724, p1 = 0.0276, ω0 = 0.024, ω1 = 1.00	-
M2a	3113.92	-	-	p0 = 0.9927, p1 = 0, p2 = 0.0073, ω0 = 0.0318, ω1 = 1.00, ω2 = 28.2246	2 (0.982); 3 (0.925); 5 (0.910); 6 (0.936)
M7	3124.70	-	-	p = 0.0569, q = 0.9137	-
M8	3114.02	-	-	p0 = 0.9927, p1 = 0.0073, p = 3.3214, q = 99.00, ω = 28.2314	2 (0.983); 3 (0.942); 5 (0.938); 6 (0.951); 100 (0.510)
M1a vs. M2a ^*b*^		17.66 *^c^*	2	-	-
M7 vs. M8 *^b^*		21.36 ^*c*^	2	-	-

*^a^* Maximum log likelihood values under specified model; *^b^* Test the relaxed selective pressure or obvious positive selection; *^c^* Significant difference.

## Supplementary Materials

The following are available online at http://www.mdpi.com/2076-2615/9/2/36/s1; Figure S1: The Hsp70 expression in the brain of the large yellow croaker based on FPKM values under hypoxia stress. Brains were harvested from six fish at the 0, 12 and 48 h time points; Table S1: Species accession numbers of Hsp70 genes in the study.
