# Supplementary material for: The Hsp70 Gene Family in Boleophthalmus pectinirostris: Genome-Wide Identification and Expression Analysis under High Ammonia Stress
Source: Animals (Basel). 2019 Jan 26;9(2):36. doi: 10.3390/ani9020036 (PMC6406738; doi:10.3390/ani9020036)
Supplement: Supplementary file 1 [file animals-09-00036-s001.pdf]

animals MDPI

Supplementary Materials: The Hsp70 Gene Family in *Boleophthalmus Pectinirostris*: Genome-Wide Identification and Expression Analysis under High Ammonia Stress

Zhaochao Deng †, Shanxiao Sun †, Tianxiang Gao and Zhiqiang Han *

Fishery College, Zhejiang Ocean University, Zhoushan, Zhejiang 316002, China; 18767788185m@sina.cn (Z.D.); jxmjsx@163.com (S.S.); gaozhang@ouc.edu.cn (T.G.)

* Correspondence: d6339124@163.com; Tel.: +86-580-2089333

† These authors contributed equally to this work.

Received: 12 December 2018; Accepted: 23 January 2019; Published: date

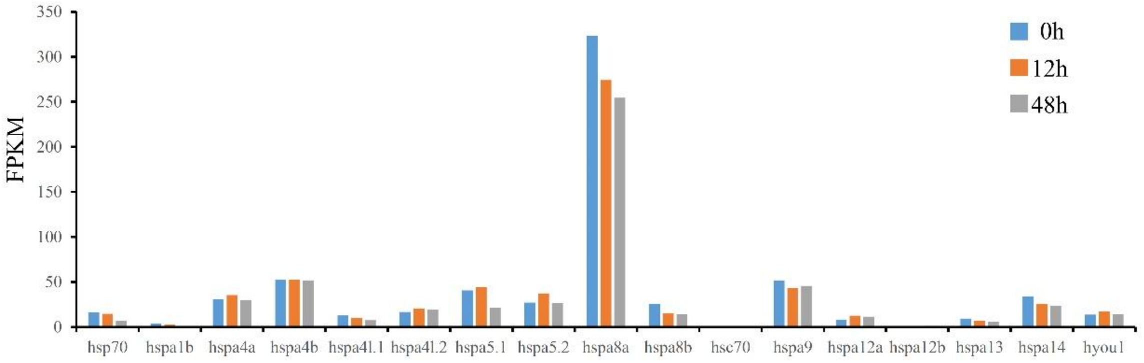

Figure S1. The Hsp70 expression in the brain of the large yellow croaker based on FPKM values under hypoxia stress. Brains were harvested from six fish at the 0, 12 and 48 h time points.

Table S1. Species accession numbers of Hsp70 genes in the study.

Associated Gene Name	NCBI/Ensemble Protein ID	Species Name	Latin name
Human_HSPA1A	NP_005336.3	human	*Homo sapiens*
Human_HSPA1B	NP_005337.2	human	*Homo sapiens*
Human_HSPA1L	NP_005518.3	human	*Homo sapiens*
Human_HSPA2	NP_068814.2	human	*Homo sapiens*
Human_HSPA4	NP_002145.3	human	*Homo sapiens*
Human_HSPA4L	NP_055093.2	human	*Homo sapiens*
Human_HSPA5	NP_005338.1	human	*Homo sapiens*
Human_HSPA6	NP_002146.2	human	*Homo sapiens*
Human_HSPA7	UniProtKB/Swiss-Prot: P48741.2	human	*Homo sapiens*
Human_HSPA8	NP_006588.1	human	*Homo sapiens*
Human_HSPA9	NP_004125.3	human	*Homo sapiens*
Human_HSPA12A	NP_079291.2	human	*Homo sapiens*
Human_HSPA12B	NP_443202.3	human	*Homo sapiens*
Human_HSPA13	NP_008879.3	human	*Homo sapiens*
Human_HSPA14	NP_057383.2	human	*Homo sapiens*
Human_HSPH1	NP_006635.2	human	*Homo sapiens*
Human_HYOU1	NP_001124463.1	human	*Homo sapiens*
Medaka_Hsp70	XP_004071143.1	medaka	*Oryzias latipes*
Medaka_Hspa1b	NP_001098384.1	medaka	*Oryzias latipes*

Medaka_Hsc70	NP_001098385.1	medaka	*Oryzias latipes*
Medaka_Hspa8b	XP_004075396.1	medaka	*Oryzias latipes*
Medaka_Hspa8a	UniProtKB/Swiss-Prot: Q9W6Y1.1	medaka	*Oryzias latipes*
Medaka_Hspa4a	ENSORLP00000001795	medaka	*Oryzias latipes*
Medaka_Hspa4b	ENSORLP00000007499	medaka	*Oryzias latipes*
Medaka_Hspa4l	XP_004082341.1	medaka	*Oryzias latipes*
Medaka_Hspa5l	XP_004074796.1	medaka	*Oryzias latipes*
Medaka_Hspa9	ENSORLP00000013340	medaka	*Oryzias latipes*
Medaka_Hspa12a	ENSORLP00000001447	medaka	*Oryzias latipes*
Medaka_Hspa12b	ENSORLP00000007349	medaka	*Oryzias latipes*
Medaka_Hspa13l	XP_004075919.1	medaka	*Oryzias latipes*
Medaka_Hspa14	ENSORLP00000015785	medaka	*Oryzias latipes*
Medaka_Hyou1l	XP_004084567.1	medaka	*Oryzias latipes*
Zebrafish_Hsp70.3	NP_571472.1	zebrafish	*Danio rerio*
Zebrafish_Hsp70.2	XP_003198158.1	zebrafish	*Danio rerio*
Zebrafish_Hsp70.1	NP_001349288.1	zebrafish	*Danio rerio*
Zebrafish_Hspa1b	NP_001093532.1	zebrafish	*Danio rerio*
Zebrafish_Hsp70l	NP_001107061.1	zebrafish	*Danio rerio*
Zebrafish_Hspa4a	NP_999881.1	zebrafish	*Danio rerio*
Zebrafish_Hspa4b	NP_956151.1	zebrafish	*Danio rerio*
Zebrafish_Hspa4l	XP_690505.2	zebrafish	*Danio rerio*
Zebrafish_Hspa5	NP_998223.1	zebrafish	*Danio rerio*
Zebrafish_Hspa8a	NP_001103873.1	zebrafish	*Danio rerio*
Zebrafish_Hspa8b	NP_001186941.1	zebrafish	*Danio rerio*
Zebrafish_Hsc70	NP_956908.1	zebrafish	*Danio rerio*
Zebrafish_Hspa9	NP_958483.2	zebrafish	*Danio rerio*
Zebrafish_Hspa12a.1	NP_001038342.1	zebrafish	*Danio rerio*
Zebrafish_Hspa12a.2	XP_003198604.1	zebrafish	*Danio rerio*
Zebrafish_Hspa12a.3	NP_001038346.2	zebrafish	*Danio rerio*
Zebrafish_Hspa13	NP_001082948.1	zebrafish	*Danio rerio*
Zebrafish_Hspa14	NP_001038541.1	zebrafish	*Danio rerio*
Zebrafish_Hsph1	XP_001919957.1	zebrafish	*Danio rerio*
Zebrafish_Hyou1	NP_997868.1	zebrafish	*Danio rerio*
Nile tilapia_Hsp70	xp_003442504.1	Nile tilapia	*Oreochromis niloticus*
Nile tilapia_Hspa1b	xp_003444871.1	Nile tilapia	*Oreochromis niloticus*
Nile tilapia_Hspa8a	xp_003448938.1	Nile tilapia	*Oreochromis niloticus*
Nile tilapia_Hsc70	xp_003454400.1	Nile tilapia	*Oreochromis niloticus*
Nile tilapia_Hspa8b	xp_003455104.1	Nile tilapia	*Oreochromis niloticus*
Nile tilapia_Hspa4l	xp_003453147.1	Nile tilapia	*Oreochromis niloticus*
Nile tilapia_Hspa5a	xp_005470418.1	Nile tilapia	*Oreochromis niloticus*
Nile tilapia_Hspa5b	xp_003459659.1	Nile tilapia	*Oreochromis niloticus*
Nile tilapia_Hspa9	xp_003459471.1	Nile tilapia	*Oreochromis niloticus*
Nile tilapia_Hspa12a	xp_003457416.1	Nile tilapia	*Oreochromis niloticus*
Nile tilapia_Hspa12b	xp_003452414.1	Nile tilapia	*Oreochromis niloticus*
Nile tilapia_Hspa13	xp_003441638.1	Nile tilapia	*Oreochromis niloticus*
Nile tilapia_Hspa14	xp_003455685.1	Nile tilapia	*Oreochromis niloticus*
Nile tilapia_Hyou1	xp_003448981.1	Nile tilapia	*Oreochromis niloticus*
Stickleback_Hspa4a	ENSGACP00000027410	Stickleback	*Gasterosteus aculeatus*
Stickleback_Hspa4b	ENSGACP00000024055	stickleback	*Gasterosteus aculeatus*
Stickleback_Hspa4l	ENSGACP00000010866	stickleback	*Gasterosteus aculeatus*
Stickleback_Hspa5	ENSGACP00000021969	stickleback	*Gasterosteus aculeatus*
Stickleback_HSPa8b	ENSGACP00000013930	stickleback	*Gasterosteus aculeatus*
Stickleback_Hspa8a	ENSGACP00000026579	stickleback	*Gasterosteus aculeatus*
Stickleback_Hspa9	ENSGACP00000025843	stickleback	*Gasterosteus aculeatus*
Stickleback_Hspa12a	ENSGACP00000019311	stickleback	*Gasterosteus aculeatus*
Stickleback_Hspa12b	ENSGACP00000026246	stickleback	*Gasterosteus aculeatus*
Stickleback_Hspa13	ENSGACP00000008388	stickleback	*Gasterosteus aculeatus*

Stickleback_Hspa14	ENSGACP00000025513	stickleback	*Gasterosteus aculeatus*
Stickleback_Hyou1	ENSGACP00000026575	stickleback	*Gasterosteus aculeatus*
